# Engineering Dirac electrons emergent on the surface of a topological insulator

**DOI:** 10.1088/1468-6996/16/1/014403

**Published:** 2015-01-16

**Authors:** Yukinori Yoshimura, Koji Kobayashi, Tomi Ohtsuki, Ken-Ichiro Imura

**Affiliations:** 1 Department of Quantum Matter, AdSM, Hiroshima University, Higashi-Hiroshima, 739-8530, Japan; 2 Department of Physics, Sophia University, Chiyoda-ku, Tokyo, 102-8554, Japan

**Keywords:** topological insulator, intrinsic Aharonov–Bohm effect, Dirac monopole, weak topological insulator, perfectly conducting channel, 71.23.-k, 71.55.Ak

## Abstract

The concept of the topological insulator (TI) has introduced a new point of view to condensed-matter physics, relating *a priori* unrelated subfields such as quantum (spin, anomalous) Hall effects, spin–orbit coupled materials, some classes of nodal superconductors, superfluid ^3^He, etc. From a technological point of view, TIs are expected to serve as platforms for realizing dissipationless transport in a non-superconducting context. The TI exhibits a gapless surface state with a characteristic conic dispersion (a surface Dirac cone). Here, we review peculiar finite-size effects applicable to such surface states in TI nanostructures. We highlight the specific electronic properties of TI nanowires and nanoparticles, and in this context we contrast the cases of weak and strong TIs. We study the robustness of the surface and the bulk of TIs against disorder, addressing the physics of Dirac and Weyl semimetals as a new research perspective in the field.

## 1. Introduction

Topological insulators [1–3] enable dissipationless transport in a non-superconducting context. They may allow for fixing ‘neutrinos’ in semiconductor chips. Known examples of the topological insulator include materials such as Bi_2_Se_3_ [4], Bi_2_Te_3_ [5], and many of their relatives [6, 7]. These are three-dimensional (3D) bulk materials under the influence of relatively strong spin–orbit coupling.

In the bulk of a sample, a topological insulator is, at least superficially, no different from a normal band insulator, but on the surface, it is quite different. A topological insulator exhibits a gapless surface state whose energy traverses the bulk energy gap. It is often said that the bulk energy gap of a topological insulator is ‘inverted’ [8, 9] in comparison with the normal one, though giving a precise meaning to this phrase needs further formulation of the bulk-effective Hamiltonian [10, 11]. Whether the gap is inverted or not is specified by a winding (topological) number that encodes the bulk band structure, and is in one-to-one correspondence with whether the system exhibits a gapless surface state (bulk-boundary correspondence) [12].

The surface state of a topological insulator exhibits a gapless spectrum often represented by the word ‘Dirac cone’, described by an (effective) two-dimensional (2D) gapless Dirac equation. Dirac electrons represented by such a Dirac equation must have a real or fictitious active spin degree of freedom. In the case of graphene, another platform for realizing such 2D Dirac electrons, this role is played by the sublattice degrees of freedom. Here, on the surface of a topological insulator, the spin is real, and since its direction is locked to the momentum (spin-to-momentum locking), the corresponding Dirac cone is often said to be *helical*. On the generically curved surface of topological insulator samples of an arbitrary shape, the same spin also locks the local tangent to the surface (spin-to-surface locking). In topological insulator nanostructures, this second type of locking, which is effective in real space, plays an important role in determining the low-energy spectrum of the surface Dirac states. In the first half of this paper, we review such finite size effects associated with the spin-to-surface locking.

Another aspect of the 3D topological insulator is that it has two subclasses: weak and strong [13–15]. The strong topological insulator exhibits a single, or more generally, an odd number of Dirac cones on its surface, while its weak counterpart exhibits an even number of Dirac cones in the surface Brillouin zone. A single Dirac cone is robust against disorder, while an even number of Dirac cones could be more easily destroyed [16, 17]. However, being robust does not necessarily equate to being *useful* [18, 19]. Recall the differences between semiconductors and metals. Metal is always conducting, while a semiconductor is either conducting or insulating, depending on the concentration of impurities. This property of the semiconductor makes it more useful than a metal, at least for some purposes, such as functioning as a transistor. The case of a weak topological insulator is somewhat similar; the fragility of the even number of Dirac cones makes the Dirac electrons emergent on the surface of a weak topological insulator controllable. In the second half of the paper, we argue that the controllability of the weak topological insulator surface states paves the way for constructing a dissipationless nanocircuit simply by patterning its surface by using lithography and etching.

## 2. Peculiar finite size effects in topological insulator surface states—part 1: the strong case

The helical Dirac electron emergent on the surface of a topological insulator is described in the 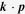 approximation by the following effective surface Dirac Hamiltonian,1
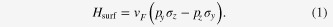
Here, the surface is chosen to be normal to the *x*-axis, and *v_F_* determine the aperture of the Dirac cone. The 2 × 2 matrix structure of equation (1) is due to the real spin degree of freedom. The form of equation (1) implies that momentum eigenstates have a spin that is oriented perpendicular to the direction of the momentum. The other way around, the spin is locked to the momentum: spin-to-momentum locking. This is the reason why the corresponding Dirac cone is said to be *helical*.

Another feature encoded in the explicit form of equation (1) is that the spin of the momentum eigenstates has no out-of-plane component. The spin is indeed locked (in-plane) to the surface. This feature, sometimes represented by the term spin-to-surface locking, also holds true on generically curved surfaces of a sample of arbitrary shape [20, 21]. Here, we will focus on cases of *cylindrical* and *spherical* shaped samples, in particular.

### 2.1. Intrinsic Aharonov–Bohm effect

Here, let us first focus on the *cylindrical* case by physically targeting a topological insulator nanowire. On the cylindrical surface of such a nanowire, equation (1) is modified as [22]2
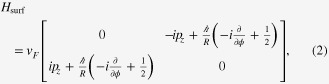
where *R* is the radius of the cylinder. The additive factor, 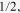 that appears in the two off-diagonals of equation (2) is a spin Berry phase encoding the constraint on the direction of spin as a consequence of the spin-to-surface locking. When a Dirac electron moves around the curved, cylindrical surface of the topological insulator nanowire, the spin-to-surface locking constrains the direction of the spin to follow the change of the tangential surface. As a result, when an electron completes a 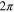 rotation in the configuration space, its spin also performs a 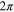 rotation in the spin space, while the spin is naturally double-valued. This explains the origin of the additive factor, 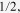 or in other words, a Berry phase, *π* [19, 20, 22, 23].

**Figure 1.**
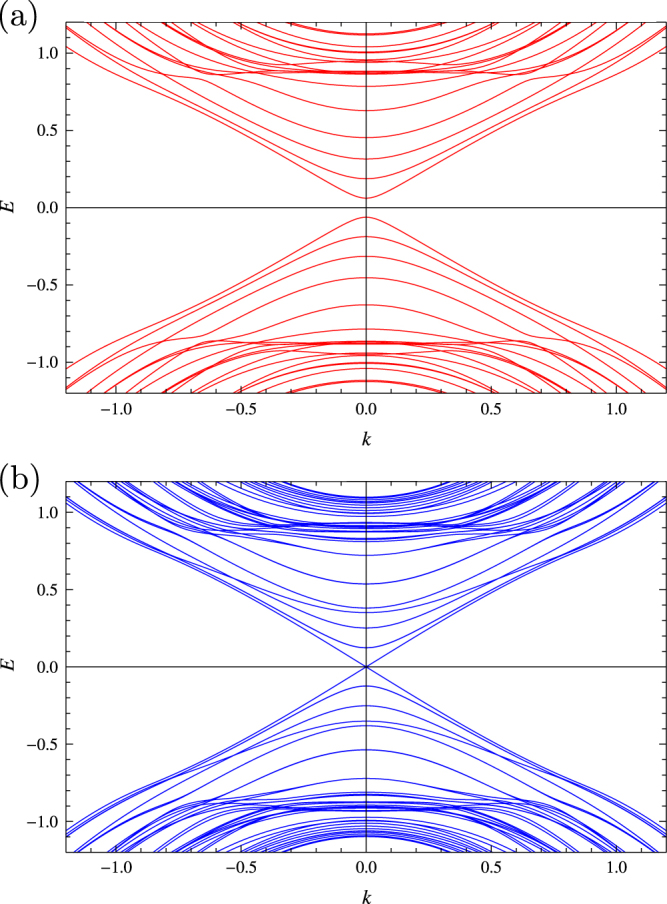
Typical energy spectrum of a rectangular, prism-shaped topological insulator nanowire. (a) As a consequence of the spin Berry phase, *π*, the spectrum of the surface state is gapped. (b) The same spectrum becomes gapless in the presence of an external flux, *π*, inserted along the axis of the prism to cancel the Berry phase.

The orbital part of an electron state on the surface of a cylindrical topological insulator is represented as3
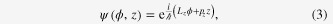
where 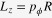 is the orbital angular momentum in the *z*-direction, along the axis of the cylinder. The Berry phase, *π*, modifies the boundary condition with respect to the polar angle, *ϕ*, from *periodic* to *anti-periodic*—that is,4
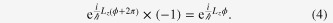
This leads to the following quantization rule of the orbital angular momentum:5
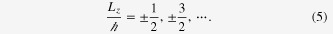
In this half-integral quantization, *L*_*z*_ = 0, and therefore 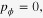 is not allowed. On the other hand, the spectrum of the surface states takes the following Dirac form,6
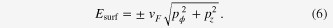
Combining equations (5) and (6), one is led to believe that the spectrum of the cylindrical topological insulator is generically *gapped*; see also a gapped spectrum shown in figure 1(a). The spectra shown in figure 1 are calculated using tight-binding implementation of the bulk 3D topological insulator of a rectangular prism shape [22].

The magnitude of this energy gap does not decrease exponentially as the size of the system increases, in sharp contrast to the case of the usual hybridization gap [24]. The energy gap, 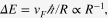 decays only algebraically as a function of the size of the system. In typical topological insulators (Bi_2_Se_3_, Bi_2_Te_3_), the Fermi velocity, 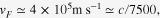 and the gap is expected to be visible in an ARPES measurement for 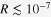 m at 1 K.

The spin Berry phase, *π*, can be interpreted in such a way that a fictitious magnetic flux is induced and pierces the cylinder. Indeed, the additive factor, 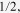 which appears in the two off-diagonals of equation (2) can be regarded as a vector potential7
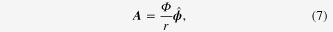
created around a flux tube of strength *Φ*, identical to half of a flux quantum, 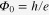. This corresponds to the Berry phase,8
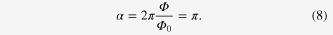



An electron on the cylindrical surface does not touch the fictitious flux itself, since the fictitious flux tube is deep inside the cylinder. Yet quantum mechanically, the spectrum of the surface state is still influenced by this effective flux; the electron feels a vector potential, and its spectrum is affected by the vector potential, even when there is no magnetic field at any position where the electron is allowed to exist (Aharonov–Bohm effect). Here, the effective flux is induced by a constraint on the spin of the surface state. Thus, the electron on the cylindrical surface induces an effective flux, while its quantum mechanical motion is influenced by the flux created by the electron itself; therefore, we call it the *intrinsic* Aharonov–Bohm effect. To confirm this scenario, we repeated the same numerical simulation in the presence of a real external magnetic flux designed to cancel the fictitious flux, as shown in equation (8). As expected, the spectrum of the surface state becomes gapless once the Berry phase, *π*, is cancelled by the external flux, as seen in figure 1(b). Related experimental results in Bi_2_Se_3_ nanowires have been reported in [25, 26].

### 2.2. Topological insulator nanoparticle as an artificial atom

Magnetic monopoles do not exist in nature. This is what we learn in courses on elementary electromagnetism, and the statement is, of course, true at the microscopic level. In matter, however, Maxwell equations are modified, or at least it is convenient to replace them with effective equations. Then, depending on the nature of the effective media, analogues of a magnetic monopole can appear. This indeed happens in the case of the spherical topological insulator.

Similarly to the cylindrical case (equation (2)), on a spherical surface of a topological insulator, equation (1) is modified to [27]9
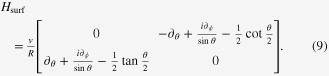
There are corrections due to the spin Berry phase in the off diagonals of equation (9). Here, in the spherical case, they are understood as vector potentials associated with a magnetic monopole. The explicit form of the vector potential differs depending on the choice of the gauge in such a way that10
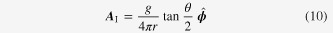
when the singularity (the Dirac string) is chosen on the 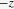 axis, while11
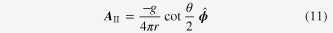
when the Dirac string is on the 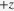 axis. Consistent with the Dirac quantization condition, equation (9) corresponds to the case of an effective magnetic monopole of strength 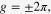 which is the smallest value compatible with the Dirac quantization condition. The electron on the surface of a *spherical* topological insulator behaves as if there is an effective *magnetic monopole* at the center of the sphere.

**Figure 2.**
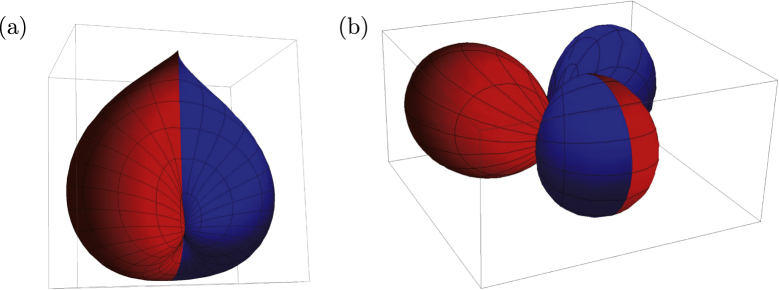
Topological insulator nanoparticle as an ‘artificial atom’. The antiperiodic version of the low-lying (a) *s*-type, and (b) *p*-type orbitals are shown. To highlight their characters, the orbitals are painted in red (in blue) when the real part of the wave function is positive (negative).

The spherical topological insulator naturally models a topological insulator nanoparticle. Taking it, therefore, as an artificial atom, let us focus on its low-lying electronic levels. The angular part of the wave function is described by an anti-periodic analogue of the spherical harmonics. A few examples are shown in figure 2, where 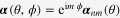 represents the angular dependence of a surface eigenfunction, specified by quantum numbers *n*, *m*. An analogue of the *s*-orbital is12
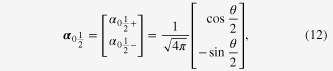
as seen in figure 2(a), while13
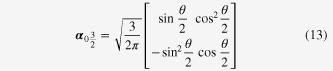
can be regarded as an anti-periodic version of the *p*-orbital, as seen in figure 2(b). Further details on such ‘monopole harmonics’ [28] and the spectrum of the artificial atom are given in [27].

## 3. Peculiar finite size effects in topological insulator surface states—part 2: the weak case

The specificity of a 3D topological insulator is that it can be either weak or strong, as mentioned in the introduction. The strong topological insulator (STI) typically exhibits a single Dirac cone, while the weak topological insulator (WTI) exhibits an even number of Dirac cones in the surface Brillouin zone. A single Dirac cone cannot be confined (Klein tunneling), while an even number of Dirac cones can be confined. Therefore, in an STI, the single Dirac cone is extended over all the surfaces. That is actually the situation we considered in section 2. In a WTI, on the other hand, some surfaces are metallic (gapless as a consequence of an even number of Dirac cones), while others are not (no Dirac cone on such surfaces).

The WTI is characterized by three weak indices: 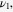 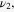 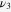 [29]. The surfaces normal to the direction specified by 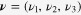 are gapped (no Dirac cone). In this sense, the WTI is by its nature *anisotropic*, and can be regarded as a *layered material*. In the following, we demonstrate that the WTI surface states are susceptible to various even/odd features in regard to the number of atomic layers stacked in the direction of 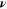.

### 3.1. Even/odd feature

**Figure 3.**
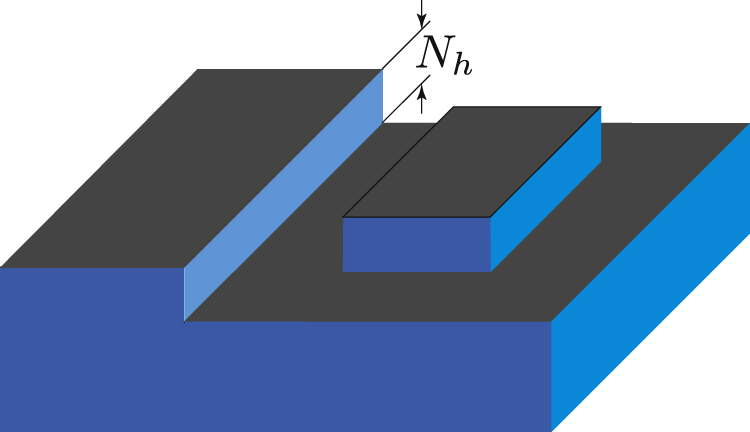
Patterning the surface of a weak topological insulator allows for constructing nanocircuits of one-dimensional (1D) dissipationless channels. See also [18].

To highlight the even/odd feature, we first consider the spectrum of a WTI sample with top, bottom, and side surfaces. The top and bottom surfaces are oriented normal to the *z*-axis. To realize a typical situation, we assume that the top and bottom surfaces are *gapped* surfaces (no Dirac cone). We then consider electronic states on a side surface, here chosen to be on the *zx*-plane. The two Dirac cones are typically located at *k*_*z*_ = 0 and at 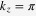. Low-energy electron states at or in the vicinity of the Dirac points may be represented by a plane wave:14
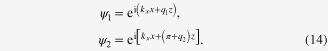
Here, the crystal momentum, *q*_1_ or *q*_2_, of the electron is measured from the corresponding Dirac cone. Now, if we consider the presence of top and bottom surfaces, located respectively at 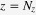 and *z* = 1 for example, we need to confine the above electron in the region of 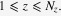 This means that we impose the boundary condition such that the wave function of the surface state, *ψ*, which may be expressed as a linear combination of 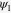 and 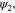 satisfies15
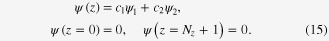
To cope with the boundary condition at *z* = 0, we choose the constants *c*_1_ and *c*_2_ such that 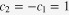. Also, at a given energy, *E*, one can set *q*_1_ and *q*_2_ such that 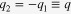. We are then left with16
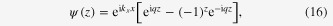
and this must vanish at 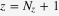. Therefore, for *N*_*z*_ odd,17
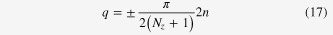
where 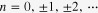 can be an arbitrary integer. Similarly, for *N*_*z*_ even, the vanishing of equation (16) at 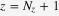 implies18
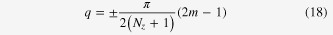
where 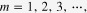 and 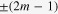 is an arbitrary odd integer. Since the spectrum of the surface state is given as [31]19
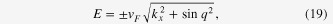
equations (17) and (18) signify that the surface spectrum is gapless when *N*_*z*_ is odd, while it is gapped when *N*_*z*_ is even [30, 31]. Also, replacing *N*_*z*_ with *N*_*h*_, one can equally apply equations (17) and (18) for characterizing the 1D helical modes that appear along a step formed on the surface of a WTI [18] (cf figure 3 and discussion given in the next section).

### 3.2. Dissipationless nanocircuit, or perfectly conducting 1D channel

We have so far argued that WTIs are susceptible to a specific type of size effect that is strongly dependent on the parity of the number of layers stacked in the direction of 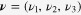. Here, we demonstrate that this even/odd feature indeed makes a WTI more useful than an STI. We consider the following Wilson–Dirac-type effective Hamiltonian on the cubic lattice,20
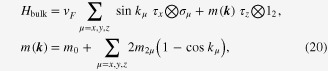
where two types of Pauli matrices, *σ* and *τ*, represent real and orbital spins, respectively, and 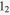 is a 2 × 2 identity matrix. We have chosen the parameters 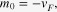 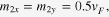 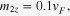 and *v_F_* = 2 so that a WTI with weak indices, 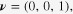 is realized [30]. By simply patterning the surface of a WTI, as seen in figure 3, one can realize a nanocircuit of a perfectly conducting 1D channel.

**Figure 4.**
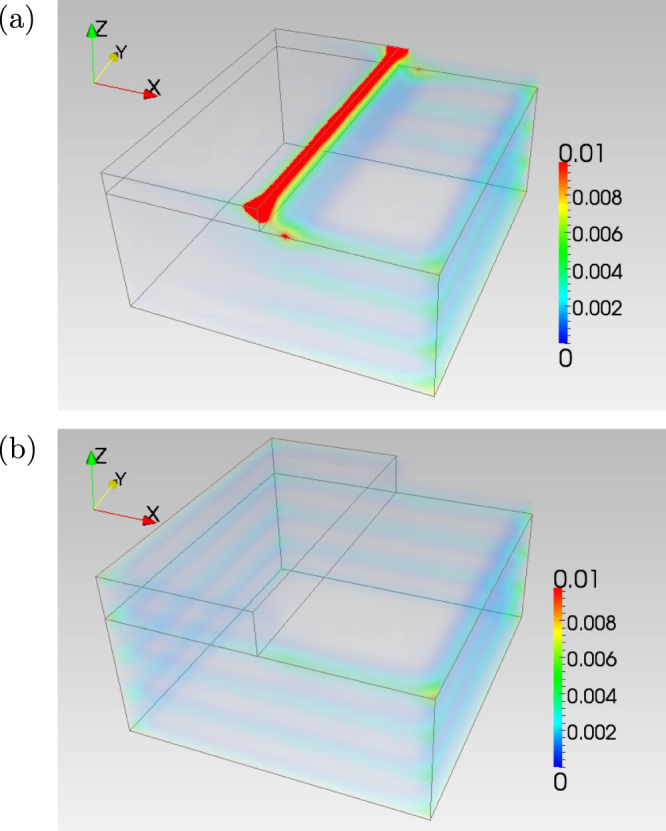
Spatial profile of low-lying electronic wave function along a step of height (a) one, and (b) two. In (a) a perfectly conducting channel is realized. See also [18].

The even/odd feature on the spectrum of a WTI sample with side surfaces is equally applied to a system with atomic scale steps on the gapped surfaces [18]. In figure 4, we show the spatial profile of the wave function of some low-lying states in a geometry with an atomic scale step. In figure 4(a), the height of the step *N*_*h*_ is 1, while in figure 4(b), the step is 2 atomic scale high. As shown in the two panels, the wave function has strong amplitude along the step when *N*_*h*_ is odd, while it has visibly no weight in the step region when *N*_*h*_ is even.

These 1D channels that appear when *N*_*h*_ is odd are also shown to be helical and robust against non-magnetic disorder [18, 32, 33]. In the supplemental material^3^ 3Wave packet dynamics in a geometry with 1, 2, and 3 atomic scale steps on top of the sample. Initial wave packet is located at *y* = 0. Parameters are the same as in figure 4 (left) without disorder and (right) with site-diagonal disorder distributed in the range [-*W*, *W*], where *W* = 1.5*v_F_* (see the online supplementary material, available at stacks.iop.org/stam/16/014403/mmedia)., we demonstrate the robustness of such an odd number of channels against disorder. Related to this, the robustness of the underlying WTI has also been studied [34, 35] (see also section 4). In any case, such a nanocircuit that appears on a patterned surface of a WTI is perfectly conducting, not only in the clean limit, but also in the presence of disorder. Therefore, a perfectly conducting 1D channel is stable, at least within the phase coherence length; to estimate the order of its magnitude, note that this is typically 200 nm for Bi_2_Se_3_ at 5 K [36].

Recently, a WTI was discovered experimentally in a bismuth-based layered bulk material, Bi_14_Rh_3_I_9_, built from grapheme-like Bi-Rh sheets [37]. Other candidates for WTIs are layered semiconductors [38], superlattices of topological and normal insulators [39–41]. The superlattice has also been considered in different contexts including as a protocol for realizing a Weyl semimetal [42], and it is indeed fabricated experimentally [43]. Still another possibility to realize a WTI-like situation is to use topological crystal insulators [44–47]. These are close relatives of the standard topological insulator, protected by crystalline symmetries instead of the time reversal symmetry.

## 4. Concluding remarks

In section 2, we highlighted the *enhanced* size effect characteristic to Dirac electrons emergent on the surface of topological insulators. In the classification of 3D topological insulators to weak and strong categories, this size effect was particularly applicable to the strong case. This type of size effect also protects the surface state from surface roughness [48]. In section 3, we discussed that weak topological insulators are susceptible to a different type of size effect that shows an even/odd feature with respect to the number of layers in the stacking direction. This even/odd feature makes *weak* topological insulators more *useful* than *strong* topological insulators. By simply patterning the surface of a weak topological insulator, one can achieve dissipationless transport in a non-superconducting context (i.e., nanocircuits of perfectly conducting 1D channels).

Although this paper has focused on the clean limit, the helical nature of the surface Dirac electron is responsible for its robustness against disorder. Because of the spin-to-momentum locking, backscattering is forbidden; for an incident state with momentum 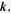 the reflected state with 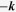 state has a spin part orthogonal to that of the initial state, leading to the absence of backscattering [49]. The gapless 2D Dirac semimetal is known to possess unusual robustness against disorder [50–52]. A slightly different question is how robust the topological classification of bulk is in the clean limit against disorder. In [34], we have addressed this issue numerically and we showed that the concept of weak and strong topological insulators remains valid at finite disorder. Then, in a more recent paper [53], we extended this study to show that both distinct topological phases and a 3D Dirac semimetal that appears at their phase boundary show some robustness against disorder. A similar result has also been obtained for a Weyl semimetal [54]. To highlight such Dirac and related Weyl semimetals, especially in the context of their robustness against disorder, is a new trend in the field, both theoretically [55, 56] and experimentally [57, 58].
